# Immune cell subsets in interface cutaneous immune-related adverse events associated with anti-PD-1 therapy resemble acute graft versus host disease more than lichen planus

**DOI:** 10.1111/cup.14242

**Published:** 2022-05-16

**Authors:** Guillermo E. Almodovar Cruz, Genevieve Kaunitz, Julie E. Stein, Inbal Sander, Travis Hollmann, Tricia R. Cottrell, Janis M. Taube, Joel C. Sunshine

**Affiliations:** 1 Department of Dermatology at Johns Hopkins University School of Medicine, Sidney Kimmel Comprehensive Cancer Center and Bloomberg-Kimmel Institute for Cancer Immunotherapy at Johns Hopkins, Baltimore, Maryland, USA; 2 Department of Pathology at Johns Hopkins University School of Medicine, Sidney Kimmel Comprehensive Cancer Center and Bloomberg-Kimmel Institute for Cancer Immunotherapy at Johns Hopkins, Baltimore, Maryland, USA; 3 Department of Pathology, Memorial Sloan Kettering Cancer Center, New York, New York, USA; 4 Department of Oncology at Johns Hopkins University School of Medicine, Sidney Kimmel Comprehensive Cancer Center and Bloomberg-Kimmel Institute for Cancer Immunotherapy at Johns Hopkins, Baltimore, Maryland, USA

**Keywords:** cutaneous eruptions, graft versus host disease, immune-related adverse events, immunologic markers, lichen planus

## Abstract

**Background:**

Checkpoint immunotherapy is frequently associated with cutaneous immune-related adverse events (cirAEs), and among those, the most common subtype shows interface reaction patterns that have been likened to lichen planus (LP); however, cutaneous acute graft versus host disease (aGVHD) may be a closer histopathologic comparator. We used quantitative pathology to compare the immunologic composition of anti-PD-1-associated interface reactions to LP and aGVHD to assess for similarities and differences between these cutaneous eruptions.

**Methods:**

Immunohistochemistry for CD4, CD8, CD68, PD-1, and PD-L1 was performed on formalin-fixed paraffin-embedded tissue from patients with anti-PD-1 interface cirAEs (*n* = 4), LP (*n* = 9), or aGVHD (*n* = 5). Densities of immune cell subsets expressing each marker were quantified using the HALO image analysis immune cell module. Plasma cell and eosinophil density were quantified on routine H&E slides.

**Results:**

Specimens from patients with anti-PD-1 interface cirAEs showed equivalent total cell densities and immune cell composition to those with aGVHD. Patients with LP showed higher total immune cell infiltration, higher absolute T-cell densities, increased CD8 proportion, and reduced histiocytic component. The cases with the highest plasma cell counts were all anti-PD-1 interface cirAEs and aGVHD.

**Conclusion:**

The composition of immune cell subsets in anti-PD-1 interface cirAEs more closely resembles the immune response seen in aGVHD than LP within our cohort. This warrants a closer look via advanced analytics and may have implications for shared pathogenesis and potential treatment options.

## 1 | INTRODUCTION

Immune checkpoint inhibitors (ICIs) have revolutionized clinical care across many tumor types. The major targeted pathways are the interaction of programmed death 1 (PD-1) with its ligand (PD-L1), and the interaction of cytotoxic T lymphocyte-associated protein 4 (CTLA-4) with B7 proteins. Both of these interactions downregulate T-cell clonal expansion, preventing the immune system from forming an adequate response to the neoantigens presented by tumor cells.^1^ Blockade of these checkpoints releases the brakes on the immune system to enhance antitumor responses.

However, in addition to unleashing antitumor responses, ICIs also produce off-target immune-related adverse events (irAEs) on other tissues.^2^ Skin, gastrointestinal, and renal tissue are the most affected by irAEs.^3^ Cutaneous toxicities are highly diverse, ranging from lichenoid dermatitis to psoriasiform eruptions, vitiligo, and immunobullous dermatoses. These cutaneous irAEs (cirAEs) often present earlier than other noncutaneous irAEs, putting dermatologists at the forefront of irAE management.^4^

Interface dermatitis is among the most common histopathologic patterns identified in cutaneous eruptions associated with PD-1 and PD-L1 blockade.^5–13^ Many cutaneous eruptions are associated with an interface pattern histopathologically. The prototypical interface dermatitis is lichen planus (LP), a mucocutaneous inflammatory disease that affects about 1% of the population. Histopathological analysis of cutaneous LP shows a dense superficial dermal bandlike infiltrate with basilar keratinocyte degeneration and pigment incontinence and is characterized by pruritic flat-topped, violaceous papules.^14^ Previous studies on interface/lichenoid irAEs suggested that some irAEs were clinically and histopathologically similar to LP or LP-like keratoses, with some noted differences including a significantly increased histiocytic component and increased spongiosis in interface reactions because of anti-PD-1 immunotherapy.^5,15–17^ In addition, molecular differences have been reported between lichenoid dermatitis irAEs and benign lichenoid keratoses, with the former exhibiting upregulation of toll-like receptor 2 (TLR2) and TLR4 and increased CD14+ and CD16+ monocytic components.^18^

Interface dermatitis also often occurs in the context of acute graft versus host disease (aGVHD), where a patient’s allogeneic hematopoietic stem cell transplant (graft) attacks the host skin to produce a rash. The strong immune response is usually driven by human leukocyte antigen mismatch, but minor histocompatibility complexes possibly play a role as well.^19^ Cutaneous manifestations of GVHD are common and may be the presenting sign of the disease.^19^ Studies have shown that post allogeneic hematopoietic stem cell transplant (allo-HST) anti-PD-1 treatment to counteract hematologic relapses has resulted in severe, and sometimes fatal, GVHD, suggesting a shared pathway between ICI treatment and the onset of GVHD.^20–22^ In addition, there is some evidence for shared mechanisms underpinning both GVHD and irAEs; for example, microRNA miR-146a is involved in both GVHD after allo-HST treatment and irAEs after ICI treatment.^23^ This suggests that, while previous studies have likened lichenoid irAEs to LP, instead interface cirAEs may be more akin to cutaneous aGVHD.

Quantitative pathology creates the opportunity for specific quantitative comparisons across disease states. The study of cutaneous irAEs may lead to an improved understanding of their pathophysiology, while also enhancing our understanding of otherwise spontaneous autoimmune diseases. In this study, we sought to use pathology to make quantitative comparisons between interface cirAEs to checkpoint inhibition and two classic interface eruptions – LP and aGVHD. To date, an extended histopathologic comparison to include aGVHD has yet to be performed.

## 2 | METHODS

### 2.1 | Case identification

**FIGURE 1:**
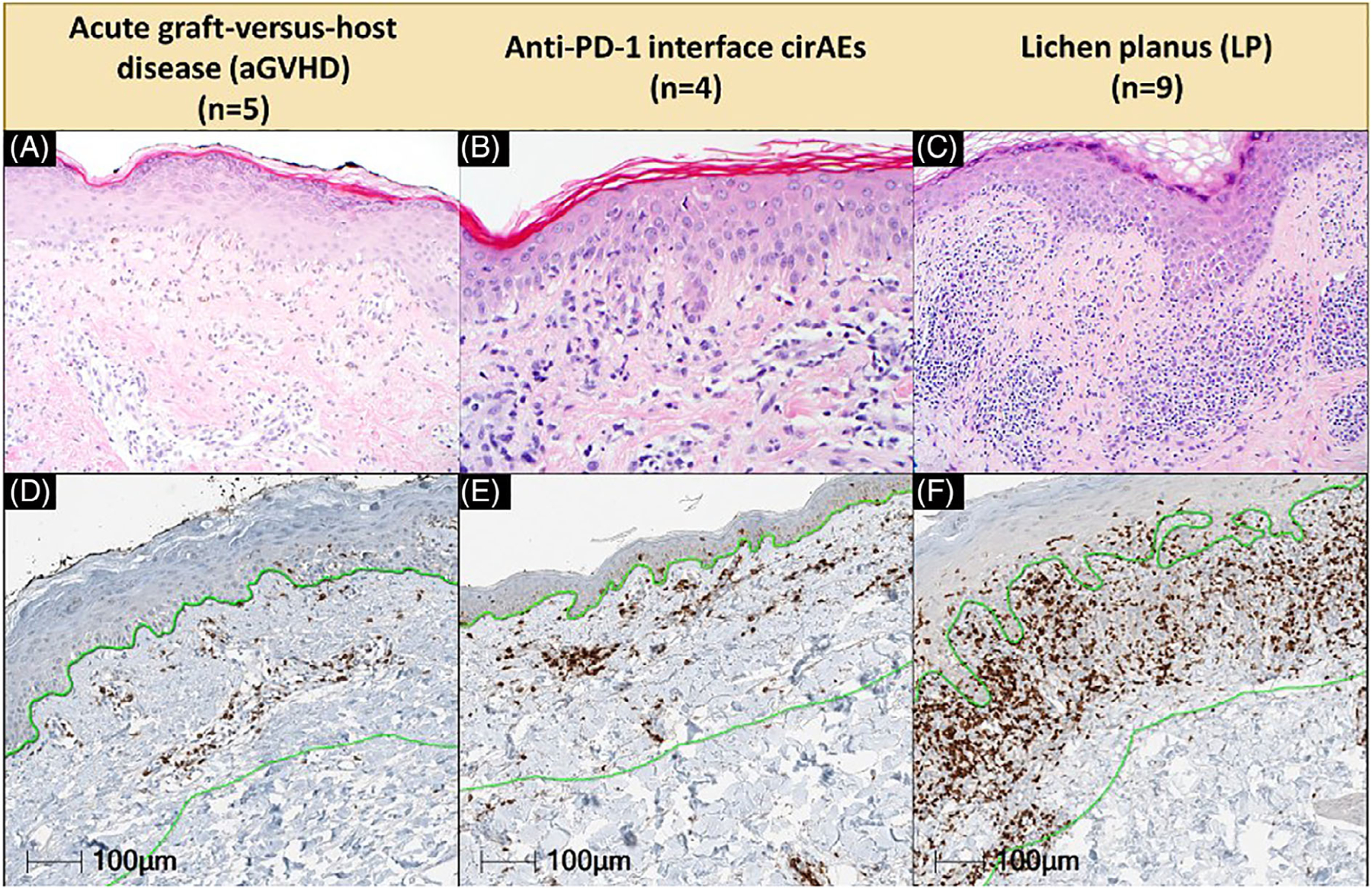
Histopathologic comparison of aGVHD, anti-PD-1 interface cirAEs, and idiopathic LP. Representative H&E (top row) and CD8 immunohistochemistry (bottom row) stained slides from patients with (A and D) aGVHD, (B and E) anti-PD-1 interface cirAEs, and (C and F) idiopathic LP. Green lines delineate annotation zone, located 300 μM from the dermal–epidermal junction (double-sided arrow). Original magnification, ×200. aGVHD, acute graft versus host disease; cirAEs, cutaneous immune-related adverse event; LP, lichen planus

Tissue specimens were identified in our Surgical Pathology archives from patients who had developed anti-PD-1 cutaneous irAEs between 2010 and 2015 and the cases were divided into interface and noninterface reaction patterns.^24^ The cases that showed an interface pattern (*n* = 4) were included in this study. In addition, over that same time period, samples from patients with cutaneous aGVHD (*n* = 5) and patients with idiopathic LP (*n* = 9) were also identified (Figure 1). For the cutaneous aGVHD cases, the timing ranged from 31 to 57 days posthematopoietic stem cell transplantation. Of the patients receiving anti-PD-1 immunotherapy, two received anti-PD-1 monotherapy, while two patients received combined anti-PD-1 and anti-CTLA-4 therapy. Additional cases from patients exhibiting the previously mentioned cutaneous eruptions were collected from the same Surgical Pathology archives between 2019 and 2021 to collect a larger cohort to measure plasma cell counts (*n* = 12, *n* = 10, and *n* = 9, respectively) and eosinophil counts (*n* = 6, *n* = 8, and *n* = 10, respectively) within tissue specimens.

### 2.2 | Immune cell quantitation

Immunohistochemistry was performed for CD4, CD8, CD68, PD-1, and PD-L1 as previously described.^25^ Absolute immune cell infiltrate densities were calculated for CD4+, CD8+, CD68+, and PD-1+ cells using the HALO image analysis immune cell module (Indica Labs), and PD-L1 was recorded as a percentage of positive cells. Infiltrate densities in anti-PD-1 interface cirAEs were compared to LP and aGVHD infiltrates. Average densities of CD4+, CD8+, and CD68+ cells were also studied by determining the relative proportion of T cells and macrophages within the tissue, and the relative compositions of each were compared between anti-PD-1 interface cirAEs, aGVHD, and LP. In addition, a total immune cell infiltrate density was calculated via summation of T-cell (CD4+, CD8+) and macrophage (CD68+) densities for comparison across all groups. H&E-stained slides were reviewed and plasma cells and eosinophils were quantified using routine H&E-stained slides by manual counting by a board-certified dermatopathologist (J.M.T.).

### 2.3 | Statistical analysis

The Mann–Whitney rank-sum test was used for comparison of total immune cell density, CD4+/CD8+/CD68+/PD-1+ cell density, proportionate CD4+/CD8+/CD68+ composition of the immune infiltrate, and plasma cell counts across disease groups. The paired Wilcoxon rank-sum test was used for comparison of CD4+ versus PD-1+ and CD8+ versus PD-1+ cell density within disease groups. *p* < 0.05 are considered significant. Statistical analyses were performed using GraphPad Prism 9.0 software (GraphPad Software Inc.).

## 3 | RESULTS

**FIGURE 2:**
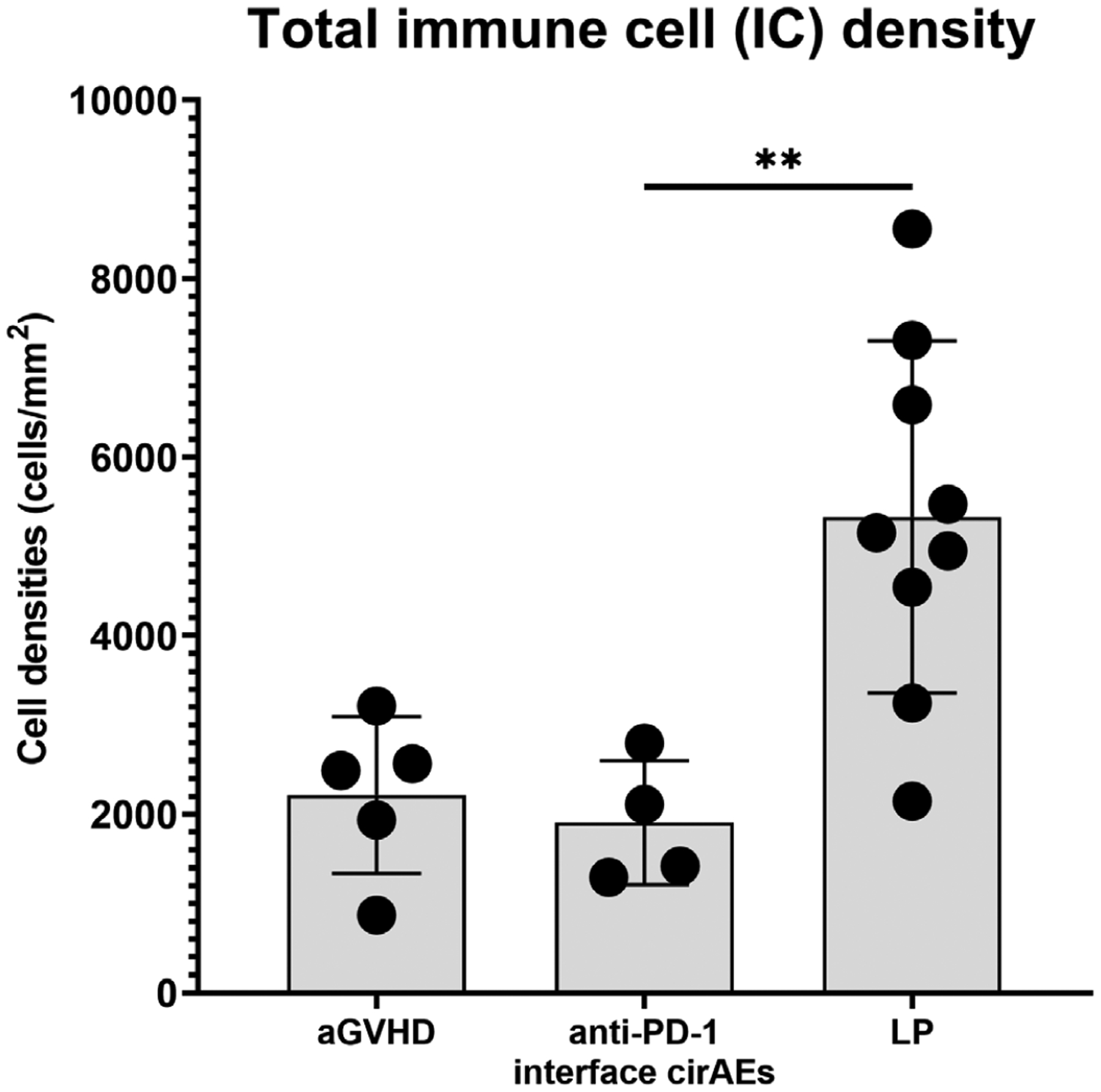
Comparison of total immune cell (IC) density between aGVHD versus anti-PD-1 interface cirAEs and LP versus anti-PD-1 interface cirAEs. Total immune cell density is calculated as the sum of CD4+, CD8+, and CD68+ cells. LP had significantly higher total immune density when compared to aGVHD or anti-PD-1 interface cirAEs (**p < 0.01, Mann–Whitney rank-sum test, error bars represent SD). aGVHD, acute graft versus host disease; cirAEs, cutaneous immune-related adverse event; LP, lichen planus

**FIGURE 3:**
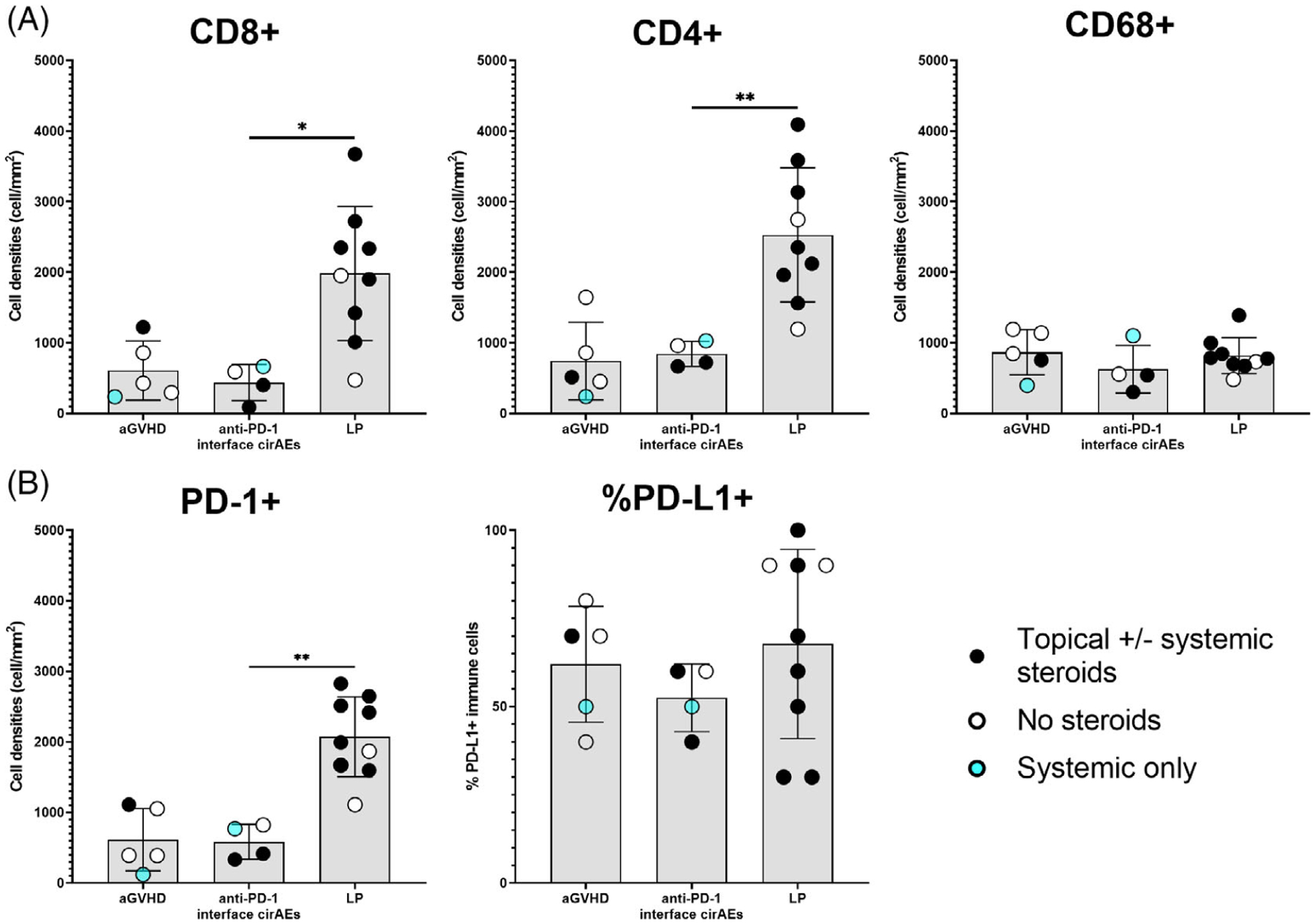
Comparison of immune cell composition and expression of immunologic checkpoints between aGVHD versus anti-PD-1 interface cirAEs and LP versus anti-PD-1 interface cirAEs. (A) LP showed significantly higher CD4+ and CD8+ T-cell densities, and equivalent CD68+ histiocyte densities when compared to anti-PD-1 interface cirAEs. (B) LP showed significantly higher PD-1 expression, and equivalent PD-L1 expression, when compared to anti-PD-1 interface cirAEs. Black circles indicate tissue samples from patients treated with topical and/or systemic steroids before biopsy; open circles designate biopsy specimens from patients not treated with steroids; blue circles represent patients who received systemic steroids only (*p < 0.05, **p < 0.01, Mann–Whitney rank-sum test, error bars represent SD). aGVHD, acute graft versus host disease; cirAEs, cutaneous immune-related adverse event; LP, lichen planus

Our analysis focused on using quantitative pathology techniques to measure the immune cell composition, PD-1/PD-L1 axis subsets, and eosinophil and plasma cell counts in patients with anti-PD-1 interface cirAEs, patients with cutaneous aGVHD, and patients with idiopathic LP. The immunologic composition of interface cirAEs to PD-1 inhibitors, aGVHD, and LP were compared by assessing CD4+, CD8+, and CD68+ cell densities. LP showed higher total immune cell infiltration than anti-PD-1 interface cirAEs, while total immune cell infiltration in aGVHD showed no significant difference from anti-PD-1 interface cirAEs (Figure 2, *p* values of 0.01 and 0.73, respectively). When comparing LP to anti-PD-1 interface cirAEs, LP cases showed higher densities of CD4+ and CD8+ cells, but not CD68+ macrophages (Figure 3A, *p* values of 0.003, 0.01, 0.26, respectively). Specifically, LP cases had an average of 2500 ± 900 CD4+ T cells/mm^2^ versus 800 ± 200 CD4+ T cells/mm^2^ for anti-PD-1 interface cirAEs and 700 ± 600 CD4+ T cells/mm^2^ for aGVHD (*p* < 0.01 for both comparisons) and an average of 2000 ± 900 CD8+ cells/mm^2^ versus 400 ± 300 CD8+ cells/mm^2^ for anti-PD-1 interface cirAEs and 600 ± 400 CD8+ cells/mm^2^ for aGVHD (*p* = 0.01 and *p* < 0.01, respectively). When we compared the immune cell densities seen in anti-PD-1 interface cirAEs and aGVHD, we found no statistically significant differences in immune cell density for CD4+ or CD8+ T cells (Figures 2 and 3A). The total density of the CD68+ histiocytic component was similar across all three cutaneous eruption types. Importantly, the use of topical or systemic steroids did not associate with decreased immune cell densities for any of the eruptions studied (Figure 3A).

**FIGURE 4:**
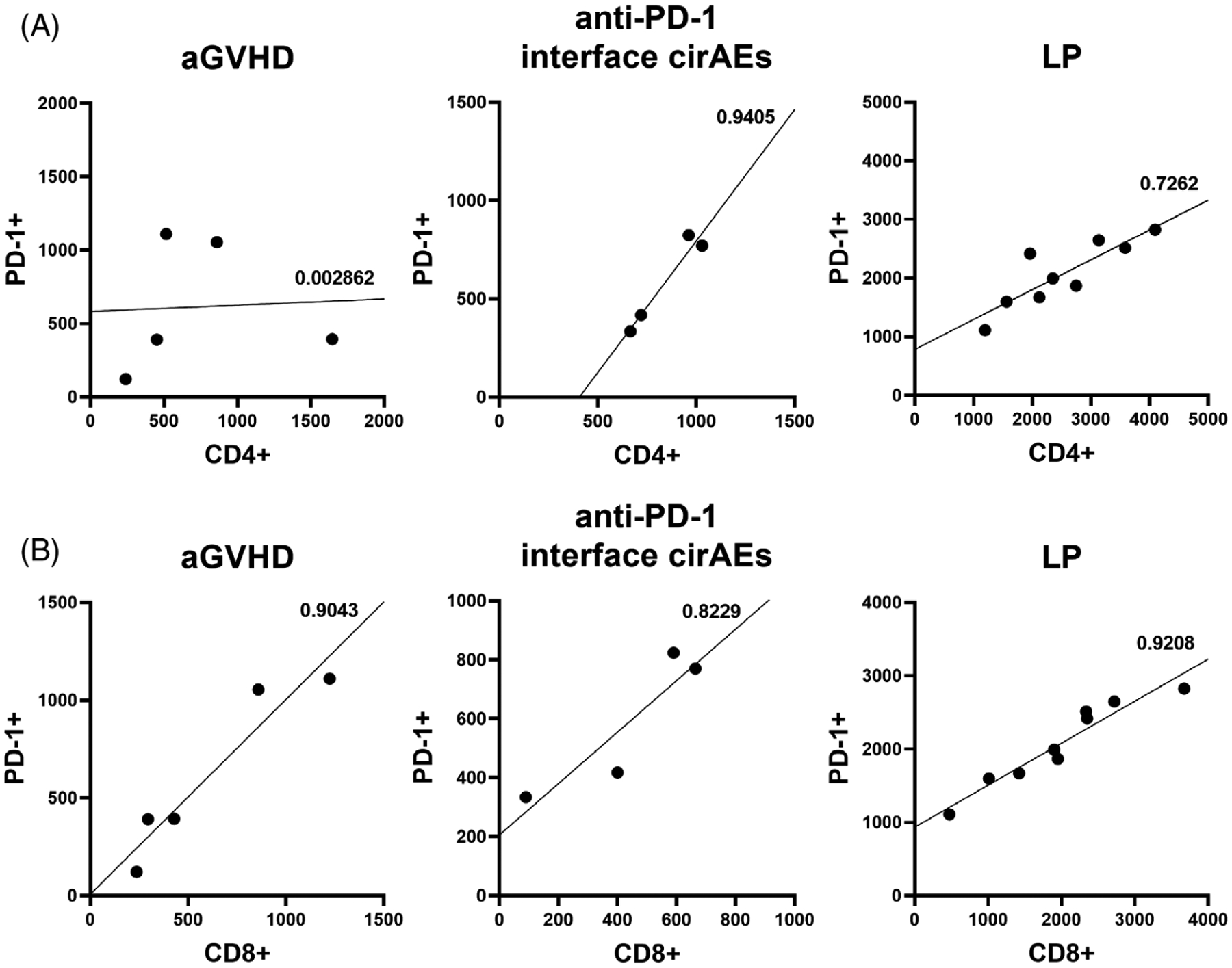
Comparison of CD4+ and CD8+ versus PD-1+ cell density in aGVHD, LP, and anti-PD-1 interface cirAEs. For the (A) CD4+ versus PD-1+ comparison, CD4+ cell densities were tracked with PD-1+ cell densities in LP and anti-PD-1 interface cirAEs, but did not track in the aGVHD patient group. For the (B) CD8+ versus PD-1+ comparisons, all three cutaneous eruption types showed PD-1+ cell densities tracked with CD8+ cell densities. *R*^2^ values are present on graphs next to the line of best fit (paired Wilcoxon rank-sum test revealed no statistical differences). aGVHD, acute graft versus host disease; cirAEs, cutaneous immune-related adverse event; LP, lichen planus

Anti-PD-1 immunotherapy interferes with the PD-1/PD-L1 immune checkpoint, helping to “release the brakes” on the immune system and encourage productive antitumor immune responses. We were interested in examining whether the expression of these critical immunologic checkpoints was altered in anti-PD-1 interface cirAEs in comparison to LP or aGVHD. PD-1+ cell density was higher in LP than in anti-PD-1 interface cirAEs, while PD-1+ cell density in aGVHD was comparable to anti-PD-1 interface cirAEs (Figure 3B, *p* = 0.003 and *p* > 0.99, respectively). The percentage of immune cells expressing PD-L1 was similar across all three eruption types (Figure 3B). As anticipated, PD-1 expression in all groups was tracked with CD8+ cell densities (Figure 4B). However, PD-1 expression was tracked with CD4+ cell densities in LP and anti-PD-1 interface cirAEs only, while it did not track in aGVHD (Figure 4A). No significant difference was seen between PD-1+ densities when compared to CD4+ and CD8+ densities within all three groups (*p* > 0.05 for all six comparisons).

**FIGURE 5:**
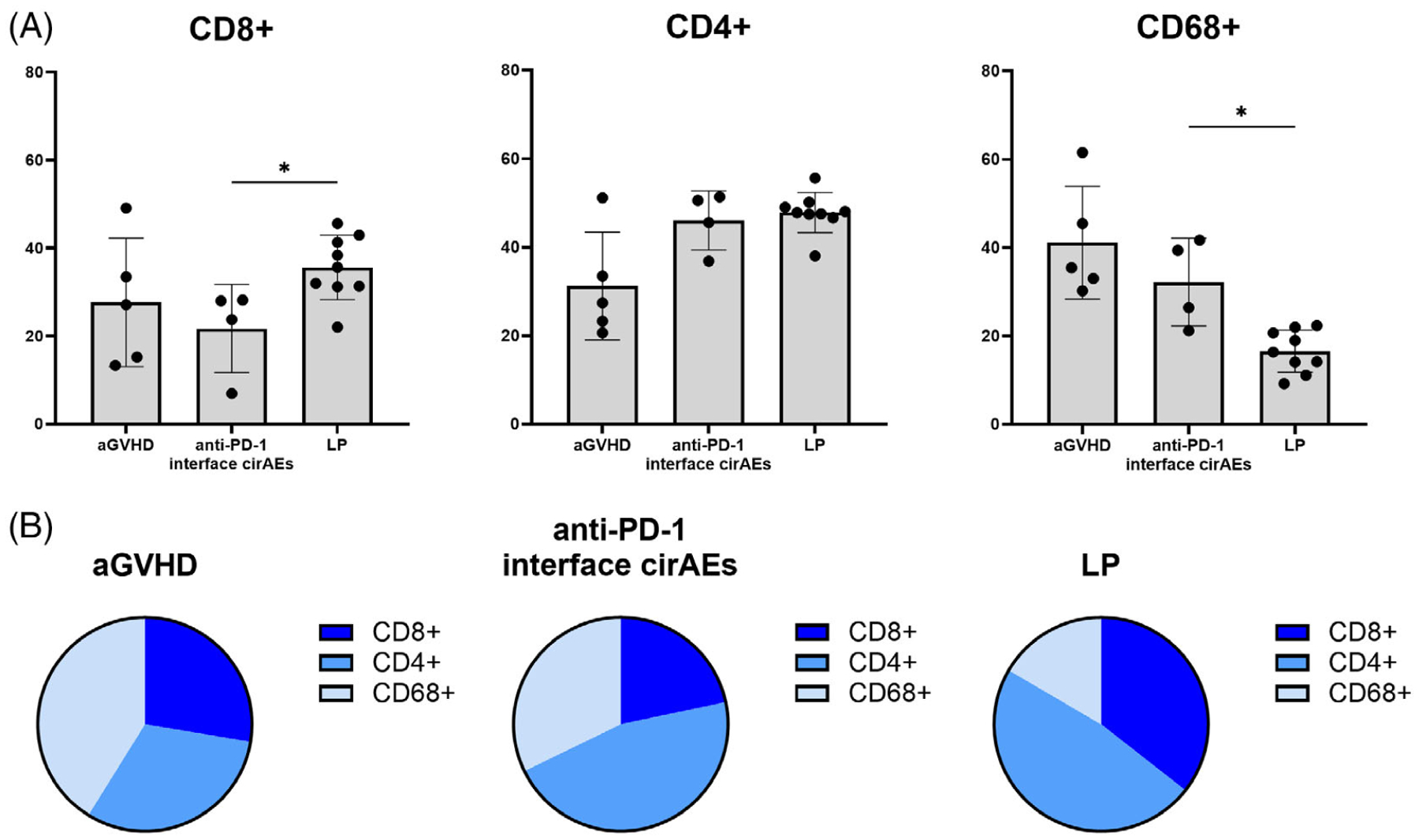
The immunologic composition of the infiltrate in anti-PD-1 interface cirAEs mirrors that of aGVHD and not LP. The relative composition of CD4+ T cells, CD8+ T cells, and CD68+ histiocytes is displayed as (A) bar charts and (B) pie charts for ease of visual comparison. LP showed an increased CD8+ fraction and decreased CD68+ histiocytic component compared to anti-PD-1 interface cirAEs (*p < 0.05, Mann–Whitney rank-sum test, error bars represent SD). aGVHD, acute graft versus host disease; cirAEs, cutaneous immune-related adverse event; LP, lichen planus

In addition to characterizing the absolute density of the infiltrates, we were also interested in examining if the relative composition of the infiltrates varied among the type of cutaneous eruptions. In this analysis, aGVHD and anti-PD-1 interface cirAEs were also similar; there was no statistical difference in relative CD8+, CD4+, and CD68+ subsets (Figure 5). LP, however, showed higher proportion of CD8+ T cells (*p* = 0.02) and a significantly smaller histiocytic component (*p* = 0.01) when compared to anti-PD-1 interface cirAEs (Figure 5).

**FIGURE 6:**
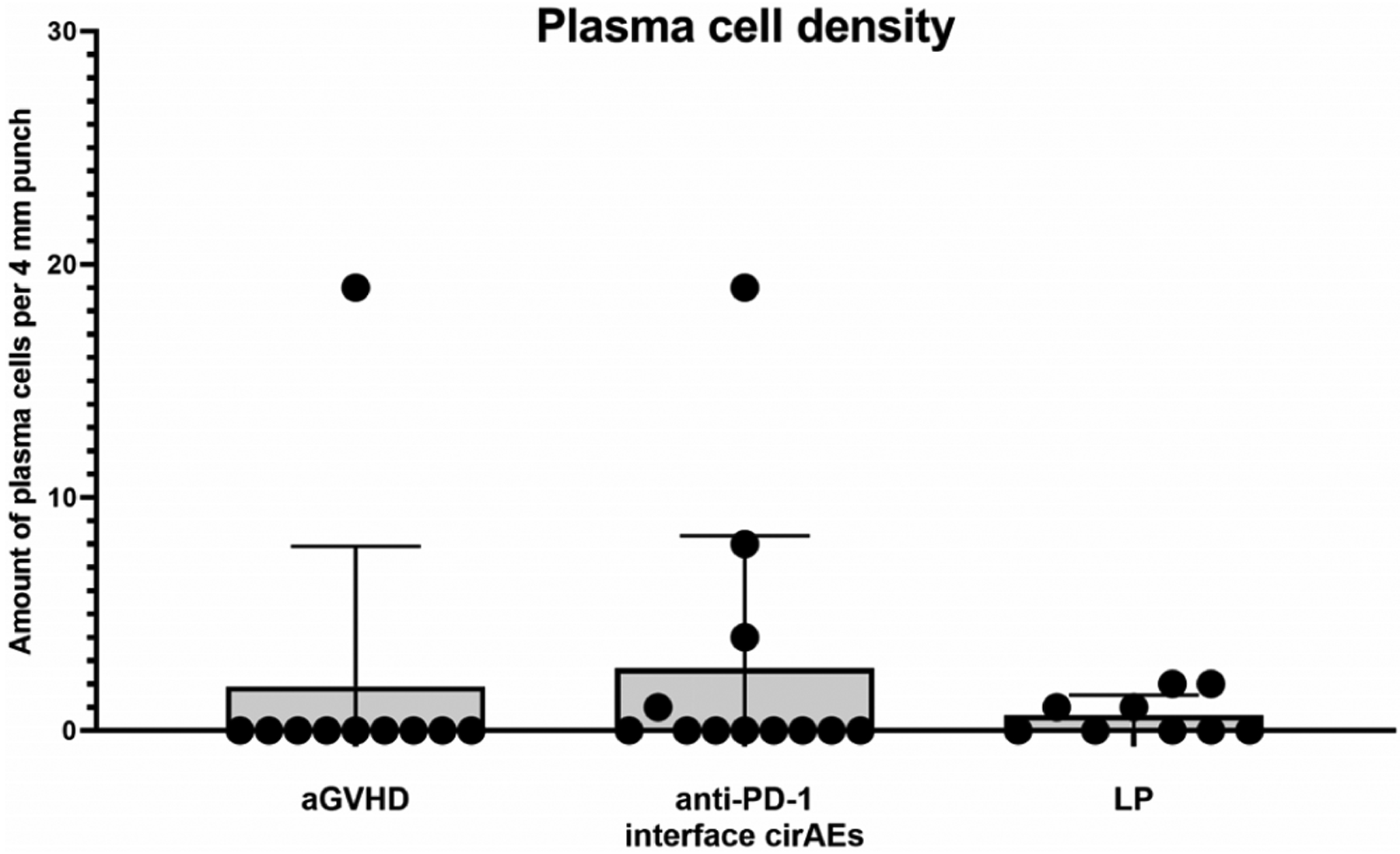
Comparison of plasma cell density between anti-PD-1 interface cirAEs, aGVHD, and LP. There were no statistically significant differences between the groups; however, the cases with increased plasmacytic component (plasma cell count >2) were either anti-PD-1 interface cirAEs or aGVHD cases (Mann–Whitney rank-sum test, error bars represent SD). aGVHD, acute graft versus host disease; cirAEs, cutaneous immune-related adverse event; LP, lichen planus

Plasma cell densities in anti-PD-1 interface cirAEs, LP, and aGVHD were also measured to identify if a relative abundance of plasma cells might offer a clue as to the identity of the eruption. In most cases, the plasma cell density was low, with 0–2 plasma cells per section, and there was no statistically significant difference identifiable in this cohort. However, the outlier cases with the most plasma cells were in anti-PD-1 interface cirAEs and aGVHD (Figure 6).

**FIGURE 7:**
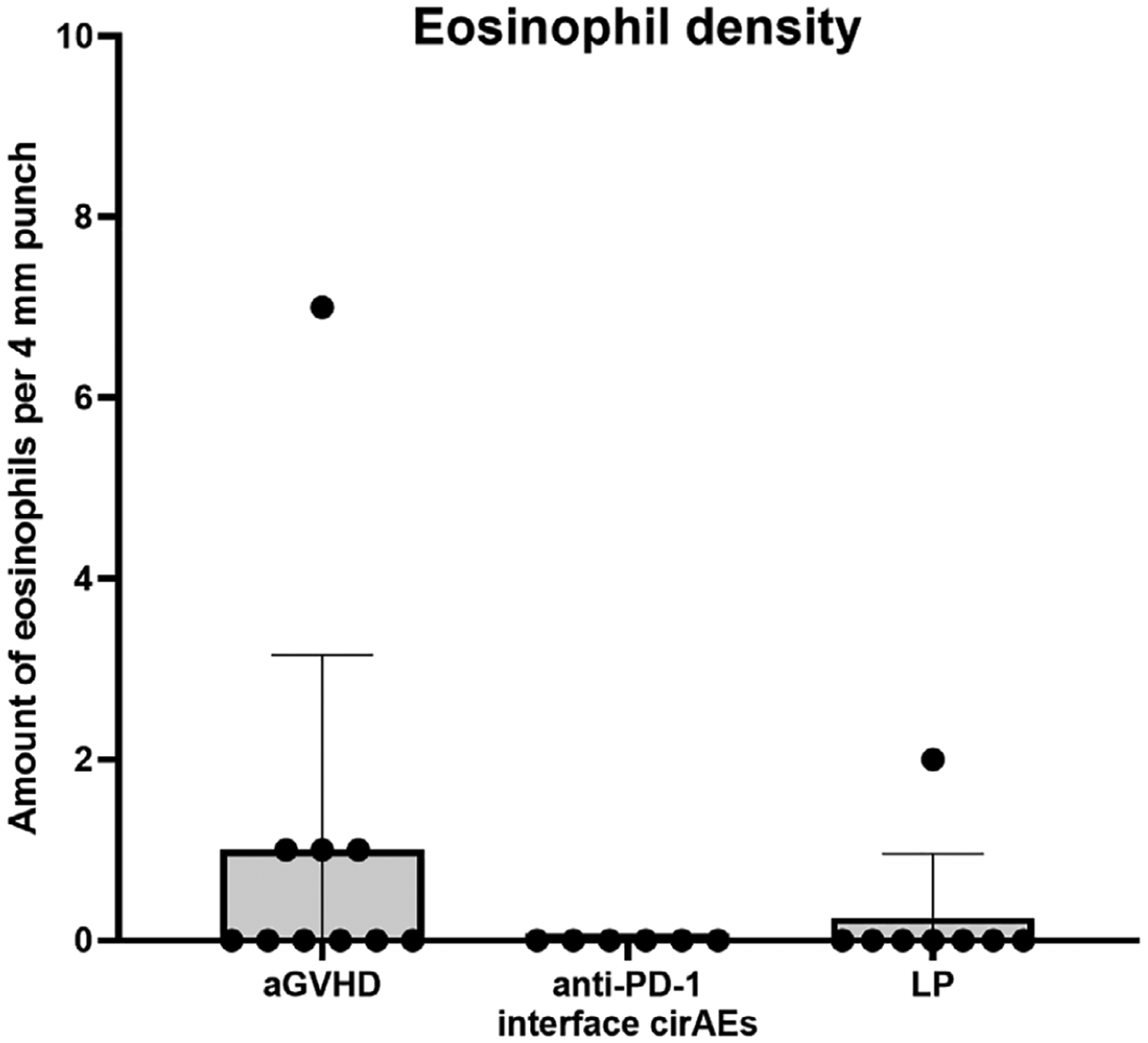
Comparison of eosinophil density between anti-PD-1 interface cirAEs, aGVHD, and LP. There were no statistically significant differences between the groups; however, the case with increased eosinophil density, equal to 7 cells per 4 mm punch, was observed in aGVHD (Mann–Whitney rank-sum test, error bars represent SD). aGVHD, acute graft versus host disease; cirAEs, cutaneous immune-related adverse event; LP, lichen planus

Eosinophil counts were also measured, and eosinophil density was not significantly different between the three disease types. In most cases, eosinophils were rare, with 0–2 eosinophils per slide. The singular outlier case with a higher eosinophil count was seen in aGVHD (Figure 7).

## 4 | DISCUSSION

Here, we present a comparison between the immune responses to anti-PD-1 interface cirAEs, LP, and aGVHD. We show that the immune response to anti-PD-1 interface cirAEs is more similar to aGVHD than to LP with respect to both total immune densities and relative composition of the infiltrates. We did not identify any statistically significant differences between cutaneous aGVHD and interface cirAEs to PD-1 inhibitors with respect to total CD4+ and CD8+ T-cell densities, CD68+ histiocyte densities, or the relative composition of T cells and histiocytes in the infiltrate. However, we did identify significant differences with respect to total immune infiltration and the relative composition of the immune infiltrate when comparing LP to anti-PD-1 interface cirAEs. We found that LP showed an increased total CD4+ and CD8+ T-cell density as compared to anti-PD-1 interface cirAEs. In addition, we found that LP showed higher CD8+ T-cell proportionality and a reduced CD68+ histiocytic proportionality as compared to anti-PD-1 interface cirAEs. Overall, we found that the immune cell subset composition is seen in interface dermatitis resulting from anti-PD-1 therapy more closely resembled the immune cell composition of aGVHD than LP within our cohort.

We also found that the presence of plasma cells is a specific but not sensitive marker for anti-PD-1 interface cirAEs. Plasma cells have been described to be a key component of immune-mediated tumor regression, suggesting that plasma cells may play a critical role in tumor clearance in the setting of checkpoint inhibition.^26,27^ In addition, PD-1 inhibitors have been shown to cause a variety of other cutaneous eruptions beyond interface eruptions; in particular, immunobullous eruptions such as bullous pemphigoid that arise as a direct consequence of inappropriate production of antihemidesmosome antibodies by plasma cells have been frequently reported, suggesting that plasma cell activation may be a component of anti-PD-1 immunotherapies.^28,29^ Plasma cells have also been previously found in lichenoid GVHD, particularly within T-cell depleted peripheral blood stem cell recipients, but less commonly in T-cell replete transplant recipients.^30^

This study highlights the phenotypic similarity between aGVHD and anti-PD-1 interface cirAEs, which may underscore a pathophysiologic connection between the two entities. PD-1 checkpoint immunotherapy functions by removing the “brakes” on the peripheral immune response. The PD-1/PD-L1 interaction is central to restraining aGVHD in both the hematopoietic stem cell transplant and solid organ transplant settings.^31–33^ Administration of anti-PD-1 inhibitors in either setting is tricky for this reason, frequently resulting in organ rejection in the solid organ transplant setting and severe and sometimes fatal GVHD postallogeneic stem cell transplantation.^32,34^

Overall, we found via quantitative pathology that anti-PD-1 interface cirAEs seem to more closely resemble the immune response seen in aGVHD as compared to LP within our small, single-institution cohorts, suggesting that there could be shared elements within the cellular pathways involved in these eruptions that should be explored further. However, our analyses are limited by the nature of standard immunohistochemistry protocols, which only allow for the staining and analysis of one biomarker per slide. This limits any study focused on the relationship between multiple biomarkers. These findings should be validated in a larger dataset as this may have implications for shared pathogenesis and potential treatment options. In addition, future directions for this work would include multiplex immunofluorescence to allow for a more in-depth look at the immune response associated with cutaneous eruptions via multiple comparisons on a single slide, allowing for coexpression analyses, and distance metrics, among other advanced analytics.^35^

## Data Availability

The data that support the findings of this study are available from the corresponding author upon reasonable request.
